# Hepato nephropathology associated with inclusion body hepatitis complicated with citrinin mycotoxicosis in a broiler farm

**DOI:** 10.14202/vetworld.2018.112-117

**Published:** 2018-02-04

**Authors:** Asok Kumar Mariappan, Palanivelu Munusamy, Shyma K. Latheef, Shambu Dayal Singh, Kuldeep Dhama

**Affiliations:** Avian Diseases Section, Division of Pathology, ICAR - Indian Veterinary Research Institute, Izatnagar, Bareilly - 243 122, Uttar Pradesh, India

**Keywords:** citrinin, fowl adenovirus, glomerulonephritis, inclusion body hepatitis, thin-layer chromatography

## Abstract

**Aim:**

Mortality in a broiler chicken farm was investigated for identifying the cause of mortality.

**Materials and Methods:**

A broiler farm with a population of 16000 succumbed to a disease outbreak. Clinical signs, vaccination history and mortality, were recorded. Necropsy examination and microscopic examination were carried out along with toxicological and molecular studies.

**Results:**

The clinical signs in the affected broiler birds were of non-specific nature with a total mortality of 26.39%. Postmortem examination and microscopical findings revealed hepatitis with basophilic intranuclear inclusion, splenitis, myocarditis, and nephritis. Glomerulonephritis was the prominent renal pathology recorded in this study. Polymerase chain reaction test confirmed the presence of fowl adenovirus (FAdV) genome in the target organs, and toxicological examination by thin-layer chromatography revealed the presence of a toxic level of citrinin in the feed samples.

**Conclusion:**

Based on various diagnostic investigations, the mortality in the flock was attributed to inclusion body hepatitis (IBH) complicated with citrinin mycotoxicosis. Thus, apart from liver pathology which occurs in a classical IBH cases, glomerulonephritis too occurs which are also a prominent finding which pathologists often miss. Thus, kidneys should also be examined histologically to assess the microscopic tissue alterations in poultry suspected for IBH along with a mycotoxicological analysis of feed. This will definitely throw light on the synergistic pathology elicited and exhibited by FAdV and mycotoxins in the poultry.

## Introduction

Poultry continues to be one of the fastest growing segments of the agricultural sector in India today. The growth rates of egg production during past 2-3 years for eggs and poultry meat are averaging at nearly 6% and 9% annually, respectively. India’s unorganized and backyard poultry sector are also one of the potent sources for subsidiary income generation by many landless/marginal farmers, and also provides nutritional security to the rural poor. There are several pathogens which affect the health and productivity of chickens, in turn causing economic losses to poultry farmers.

Fowl adenovirus (FAdV) infection in broilers is one such disease which causes substantial economic losses to poultry farmers by causing mortality and production loss due to the poor performance of the chickens [1]. They cause different disease entities, namely, inclusion body hepatitis (IBH), hydropericardium syndrome (HPS), respiratory infections, gizzard erosions, arthritis, and pancreatitis. Apart from its direct effect, the virus itself is an immunosuppressive agent which leads to secondary complications. Based on the worldwide distribution and ubiquitous presence of adenoviruses in healthy poultry flocks, most of the FAdVs are considered to be non-pathogenic; however, some Group I FAdVs, including serotype 4 and 8, appears to be pathogenic and are associated with clinical manifestations of IBH in chickens [2]. Earlier, it was suggested that immune suppression due to pre-infection or concurrent infection with infectious bursal disease virus (IBDV) or chicken anemia virus (CAV) induces IBH in chickens. However, recent studies suggest that IBDV and CAV infections or other immunosuppressive agents may not be essential factors needed for the onset of IBH caused by FAdV in chickens, which indicates that FAdV is evolving as a primary pathogen causing significant diseases [3] with or without any predisposing factors. Mycotoxicosis caused by citrinin, a nephrotoxic mycotoxin, is produced by molds including *Monascus* species (*Monascus purpureus* and *Meiothermus ruber*) and *Penicillium* species such as *Penicillium citrinum*, *Penicillium expansum*, *Penicillium radicicola*, and *Penicillium verrucosum* [4,5], and causes severe nephrotic changes in poultry species [6]. Apart from its nephrotoxicity, citrinin is a potent immunotoxic, neurotoxic, embryotoxic, and teratogenic mycotoxin in different animal species [7,8]. Pathological changes due to nephrotoxicity include degenerative and necrotic changes affecting the renal tubular epithelial cells. Hepatomegaly in citrinin toxicoses do occur mainly due to hepatic degeneration and sinusoidal congestion [9]. Liver is involved in a diverse range of essential functions that includes storage, digestion, absorption, and metabolism of nutrients and toxins from both the portal and systemic circulation, and the synthesis of carbohydrates, clotting factors, vitamins, and other proteins. It is one of the major organs involved in detoxification of toxic metabolites, plays an important role in eliminating toxic materials from blood and also helps in the destruction of degenerated red blood cells.

The pathological alterations of liver in poultry are multifactorial in origin and/are a common problem observed in many infectious/non-infectious diseases. Besides liver, kidney also plays a major role in detoxification of toxin such as citrinin by possessing abundant cytochrome P450 enzyme expression [10]. Thus, kidney dysfunction alike hepatic problems succumb birds to production loss. There are several pathological conditions which affect kidneys, and glomerulonephritis is noteworthy to be mentioned. Glomerulonephritis associated with IBH and citrinin toxicity are rarely reported in broiler chickens. Thus, any pathological conditions affecting both liver and kidney will hamper the production and productivity of the poultry. In this study, we describe detailed pathological and molecular investigation, in an organized farm, of IBH and citrinin toxicity in broiler chickens.

## Materials and Methods

### Ethical approval

The approval from the Institutional Animal Ethics Committee to carry out the current study was not required as the samples were from dead birds brought for Postmortem examination.

### Farm history, necropsy examination, and collection of samples

A disease outbreak in an organized poultry farm in Bareilly region was investigated (n=16000). Daily Mortality of birds was recorded. A total of 4223 (26.39%) birds died during the outbreak. Clinical signs, vaccination history, and mortality were recorded. The birds were vaccinated against Newcastle disease and infectious bursal disease. Necropsy examination of the dead birds was carried out, and the gross findings were recorded. Representative tissue samples were collected and stored in 10% formalin for histopathological studies and in −80°C for molecular studies.

### Histopathology

The tissue samples were fixed in 10% neutral buffered formalin and processed by routine paraffin-embedding technique. Briefly, 4-5 µm thick sections were deparaffinized and stained by Hematoxylin and Eosin staining method for detailed microscopic studies [11].

### Genome detection studies (DNA isolation, polymerase chain reaction [PCR], and electrophoresis)

The tissue samples stored in −80°C were subjected to DNA extraction with DNeasy blood and tissue kit (Qiagen, Germany) as per the manufacturer’s instructions. PCR was performed with the primers targeting the gene encoding L1 region of the hexon protein [12] of FAdV Group I (forward primer, 5’– CAARTTCAGRCAGACGGT –3’; reverse primer 5’– TAGTGATGMCGSGACATCAT –3’). The PCR was performed in a 25 µL reaction mixture containing 1.0 µL DNA (5 ng/uL), 12.5 µL DreamTaq PCR Master mix 2× (Thermo Scientific, USA), 1.0 µL forward primer (10 p mol/µL), 1.0 µL reverse primer (10 p mol/µL), and 7.5 µL nuclease-free water. The cyclic conditions for primary amplification were initial denaturation at 95°C for 15 min, followed by 35 cycles of 94°C for 45 s, 57°C for 45 s, 72°C for 45 s, and final extension at 72°C for 10 min. The PCR products were analyzed by electrophoresis in 1.5% agarose gel (in 0.5× Tris-Borate-EDTA buffer) stained with ethidium bromide (0.5-µg/mL). After sufficient migration of the dye, the gel was visualized in an ultraviolet- transilluminator (302-nm) and documented by alpha imager software. The relative size of the amplified product was determined by comparison with standard DNA molecular weight markers run along with the PCR products (Thermo Scientific, USA).

### Toxicological analysis of feed sample and organs

Feed samples and organs (liver and kidney) collected during postmortem examination were sent to Toxicology lab, Centre for Animal Disease Research and Diagnosis, ICAR-IVRI, Izatnagar, Bareilly, for mycotoxin analysis.

## Results

### Mortality and clinical signs

Daily mortality of 30-40/day started at 14 days post-hatching and the daily mortality peaked to 40-50/days at 25 days of age. Post-intervention with the antibiotics and feed change, the mortality subsided. The clinical signs in the affected broiler birds were of non-specific nature including depression, ruffled feathers, anorexia, inappetence, reduced water intake, huddling, smothering, and prostration followed by death. During the course of the disease, total mortality reached 26.39%.

### Gross findings

**Figure-1:**
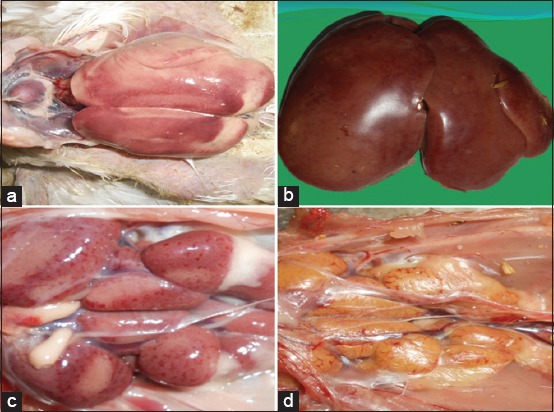
(a) Swollen and pale liver with multifocal necrotic foci over the surface, (b) swollen and icteric liver, (c) swollen and edematous kidneys with multifocal pinpoint petechial hemorrhages, (d) swollen and icteric kidneys.

The birds were stunted and had poor body condition. The conjunctival mucous membrane was pale and icteric in few cases. Facial swelling with mucoid to catarrhal exudate from the nares was evident in few cases. The lungs were pneumonic. The main lesions in necropsied birds were pale/icteric, friable, and swollen livers (Figure-1a). Liver also contained numerous focal to coalescing pale necrotic areas (Figure-1b). Petechial or ecchymotic hemorrhages were noticed in the liver and skeletal muscles (thigh and breast muscles). The intestines revealed severe mucosal congestion with excess catarrhal exudate in the lumen. Pancreas was swollen and pale. Mottling of spleen was evident and characterized by numerous multifocal pale areas on the surface. Kidneys were swollen pale (Figure-1c) and icteric (Figure-1d) in few cases and showed a prominent gross tubular pattern. In few cases, subcapsular hemorrhages were evident in kidneys. The bursa of Fabricius was edematous. Fat necrosis of the abdominal fat giving a cooked up appearance was recorded.

### Histopathological findings

**Figure-2:**
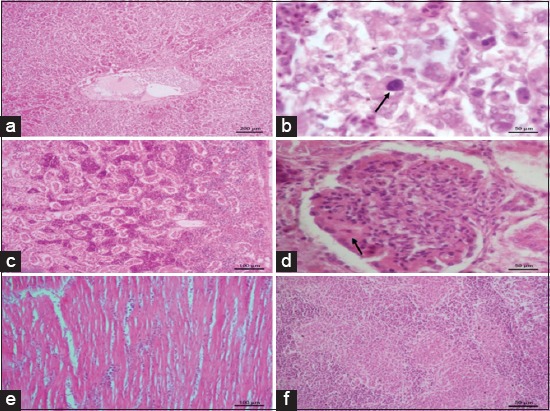
(a) Loss of normal architecture of hepatocytes with coalescing necrosis, (b) large basophilic intranuclear inclusion bodies in the hepatocyte with clear halo (arrow), (c) kidneys are showing subcapsular hemorrhages, hyperemia, denuded tubular epithelium, (d) swollen and hypercellular glomeruli with eosinophilic homogenous material deposition around the glomerular tufts (arrow), (e) myocarditis with degenerated muscle fibers and interstitial lymphocytic infiltration, (f) splenic parenchyma showing multifocal areas of lymphoid depletion and associated reticular endothelial cell hyperplasia.

On histopathological examinations, significant lesions were seen in the liver, kidney, heart, and spleen. Loss of normal architecture of hepatocytes was evident (Figure-2a). The hepatocytes showed both degenerative and necrotic changes. Varying degrees of degeneration, namely, cloudy swelling, vacuolar degeneration, and fatty changes with peripherally arranged nuclei were evident. Multifocal areas of coagulative necrosis were consistent in liver. Large irregular basophilic intranuclear inclusion bodies were seen in hepatocytes (Figure-2b). Kidneys showed subcapsular hemorrhages (Figure-2c), hyperemia, denuded tubular epithelium, tubular epithelial degeneration, necrosis, and mild interstitial lymphoplasmacytic nephritis. Another prominent finding was seen in glomeruli, the affected glomeruli were swollen, hypercellular and had deposition of eosinophilic homogenous material around the glomerular tufts (Figure-2d). Mild focal myocarditis characterized by degenerated muscle fibers and interstitial lymphocytic infiltration was seen in the heart sections (Figure-2e). Splenic parenchyma revealed multifocal areas of lymphoid depletion and associated reticular endothelial cell hyperplasia (Figure-2f).

### Genome detection studies

**Figure-3:**
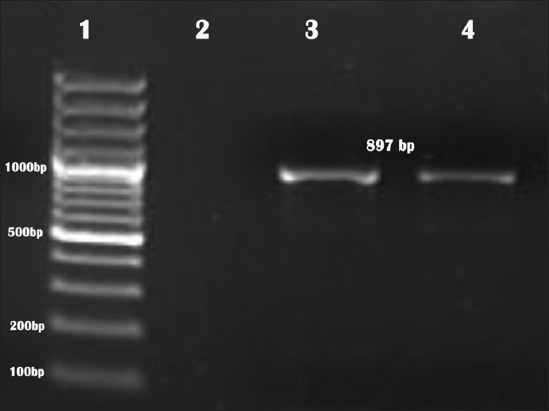
Agar gel electrophoretic picture showing 897 bp polymerase chain reaction-amplified product of hexon gene specific for fowl adenovirus (1 - DNA ladder; 2 - non template control; 3 - positive control, 4 - test sample).

An amplicon of size 897 bp on 1.5% agarose gel was visualized, indicating the presence of FAdV genome in the samples (Figure-3).

### Toxicological analysis of feed sample and organs

Feed and organ samples were positive for citrinin. The quantification of citrinin was done using thin-layer chromatography (TLC), and it was found to be 2.0 ppm in feed.

## Discussion

Chickens in a broiler farm succumbed to severe mortality despite routine vaccination regime. Prominent gross lesions observed were severe hepatomegaly, splenomegaly, nephromegaly, and icterus. Initially, the molecular investigation was undertaken to investigate the involvement of FAdV using PCR, which confirmed the presence of FAdV in the tissues. The gross lesions observed in the present study were in terms with classical lesions reported earlier in case of IBH [13]. FAdV causes IBH and other different disease entities, namely, HPS, reduced egg production, tenosynovitis, impaired growth, aplastic anemia, atrophy of bursa and thymus, enteritis, and conjunctivitis in poultry and other birds [12,14]. In case of IBH, mortality varies between 10% and as high as 50% depending on the strain of virus and host susceptibility [13,15]. The mortality rate recorded in the present investigation was 26.39%</AQ5>. The IBH can affect all ages of broiler, and the disease has been diagnosed in young chicks of even 4 days age [16, 17]. There exists an inverse relationship between the age of the host and the clinical disease by FAdV, wherein there is a reduction in mortality as the age of the bird advance which has been attributed to the restriction of viral replication inside the host cells [17,18]. The above findings are in corroboration with the present study wherein the peak mortality was seen at 14 days and then there was a drop in mortality rate after therapeutic intervention with antibiotic treatment and feed change as the bird advanced to 25 days.

Mere detection of FAdV in tissues is not considered as diagnostic importance as the viral genome can be detected in both sick and healthy birds because of its ubiquitous nature [3,19] thus further conventional Hematoxylin and eosin staining of liver sections was done to confirm the lesions of IBH microscopically. Pathognomonic intranuclear basophilic inclusion bodies could be detected with associated degenerative and necrotic changes in the liver sections. Thus, detection of intranuclear inclusion bodies confirmed the role of FAdV in causing IBH in the chicken flock which was also reported by earlier workers [3,20,21].

Before FAdV infection, priming by certain immunosuppressive diseases/conditions such as chicken infectious anemia, IBD, and mycotoxicosis enhances the pathogenicity of IBH [22,23]. To rule out mycotoxins in the feed, the feed and tissue samples were sent for mycotoxin detection. The TLC analysis indicated the presence of 2 ppm citrinin in the feed samples tested. Literature survey on adverse effects of citrinin in poultry revealed only fewer studies. Broiler chicks showed moderate toxic effects when fed with 1.7 mg/kg body weight [24]. Abdelhamid and Dorra [25] reported that laying hens administered with citrinin of 6.25 µg/kg b.w. per day showed toxic effects. On the contrary to the above findings, other studies revealed toxic effect at comparatively higher doses only. Thus, it was documented that toxic effects in poultry varied based on species, age and experimental design [26]. The enhancing effect of pathogenicity of IBH could be attributed to the immunosuppressive effect of mycotoxins. In a study by Singh *et al*. [27] citrinosis was proved to immunosuppression by decreasing cell-mediated immunity, depletion of lymphocytes in the spleen and Peyer’s patches and also a severe degree of lymphocytopenia. Later, as per studies byShivachandra *et a*l. [28], the workers had proved that administering mycotoxins enhances FAdV virulence by causing immunosuppression. Similar results of citrinin mediated immunosuppression have also been documented [29]. The citrinin toxicity causes primary changes in the kidney characterized by severe degenerative and necrotic changes in the renal tubular epithelial cells causing severe diuresis A prominent finding recorded in the present investigation was membranoproliferative glomerulonephritis wherein the affected glomeruli were swollen, hypercellular and there was eosinophilic homogenous material deposited around the glomerular tufts. Membranoproliferative glomerulonephritis has been reported in broiler chickens associated with IBH and citrinin toxicity [30,31]. Furthermore, neoplasia, autoimmune disorders, and persistent infections due to prolonged viral and bacterial stimulation can cause membranoproliferative glomerulonephritis [32]. It has been reported that glomerular lesions occurring in viral infection are mainly due to immune complex deposition [30]. In citrinin toxicity, inability to filtrate citrinin bound serum proteins and albumin leads to immune complex-induced nephritis [31]. Apart from citrinin induced nephropathology, citrinin is reported to be a potent immunosuppressive agent due to lymphocytolysis and a potent hepatotoxic agent [7,8,33]. There could be a synergistic effect of FAdV and citrinin on liver and kidney pathology; however, interaction between citrinin and FAdV in causing hepato nephropathology needs further research.

Based on the gross, microscopical findings and genomic detection studies the mortality in the investigated chicken flock was due to IBH complicated with citrinin mycotoxicosis. To conclude, apart from liver, kidney should also be examined histologically to assess the microscopic details in cases of IBH, and it is important to carry out mycotoxicological analysis of feed and tissue samples. This will definitely throw light on the synergistic pathology exhibited by FAdV and mycotoxins in the poultry. The above strategy will definitely help in designing suitable control strategies when FAdV is suspected in a disease outbreak which in turn reduces the economic losses to the poultry farmers.

## Authors’ Contributions

AK contributed in conception/design of the work, data collection, data analysis and interpretation, drafting the article, critical revision of the article and final approval of the version to be published. PM helped in data analysis and interpretation, drafting the article, critical revision of the article and final approval of the version to be published. SK did data analysis and interpretation, drafting the article and critical revision of the article. SDS helped in critical revision of the article. KD helped in critical revision of the article and final approval of the version to be published. All authors read and approved the final manuscript.
